# Nurses and midwives demographic shift in Ghana—the policy implications of a looming crisis

**DOI:** 10.1186/s12960-019-0377-1

**Published:** 2019-05-22

**Authors:** James Avoka Asamani, Ninon P. Amertil, Hamza Ismaila, Akugri Abande Francis, Margaret M. Chebere, Juliet Nabyonga-Orem

**Affiliations:** 1 World Health Organization, Regional Office for Africa, Inter-Country Support Team for Eastern and Southern Africa, Harare, Zimbabwe; 2 School of Nursing and Midwifery, Valley View University, Oyibi, Accra, Ghana; 3 Human Resources Division, Ghana Health Services, Accra, Ghana

**Keywords:** Human resources for health, Nursing and midwifery workforce, Nursing demographics, Health workforce policy

## Abstract

As part of measures to address severe shortage of nurses and midwives, Ghana embarked on massive scale-up of the production of nurses and midwives which has yielded remarkable improvements in nurse staffing levels. It has, however, also resulted in a dramatic demographic shift in the nursing and midwifery workforce in which 71 to 93% of nurses and midwives by 2018 were 35 years or younger, as compared with 2.8 to 44% in 2008. In this commentary, we examine how the drastic generational transition could adversely impact on the quality of nursing care and how the educational advancement needs of the young generation of the nursing and midwifery workforce are not being met. We propose the institution of a national nursing and midwifery mentorship programme and a review of the study leave policy to make it flexible and be based on a comprehensive training needs assessment of the nursing and midwifery workforce. We further advocate that policymakers should also consider upgrading all professional nursing and midwifery programmes to bachelor degrees as this would not only potentially enhance the quality of training but also address the phenomenon of large numbers of nurses and midwives seeking bachelor degree training soon after employment—sometimes putting them at the offending side of organisational policy.

## Introduction

The global community is increasingly recognising the contribution of nurses and midwives to health service delivery and the need to harness the nursing and midwifery potential towards the attainment of Universal Health Coverage (UHC) and the Sustainable Development Goals (SDGs) especially goal 3. In leaving no one behind as enshrined in the UHC effort, nurses and midwives who undoubtedly have been the bedrock of most healthcare systems and form the bulk of the health workforce [1, 2] would have to be a greater part of the efforts.

The 2006 World Health Report classified Ghana among 36 countries in sub-Saharan Africa facing Human Resources for Health (HRH) crisis [3] which became a clarion call for concerted efforts to address a myriad of health workforce challenges notably, inadequate production, excessive out-migration and low wages among others. Consequently, the Ministry of Health developed the HRH strategy, 2007–2011 [4, 5] which outlined a number of strategic interventions including the expansion and liberalisation of the training of health workers, the reintroduction of auxiliary nursing programmes, the establishment of postgraduate specialist training colleges (for doctors, pharmacist, nurses and midwives), review of health workers salary structure and the introduction of staff vehicle hire purchase scheme, to name a few. It would appear that these efforts to a large extent yielded some fruits as the density of doctors, nurses and midwives dramatically improved from 1.07 per 1000 population in 2005 to 2.65 per 1000 population in 2017 [6]. These efforts have not only resulted in improved service coverage towards UHC, but also the country is being cited as a leading producer of physicians, nurses and midwives in sub-Saharan Africa [7, 8].

Out of about 115 650 health workers employed in the public sector in 2018, 58% were nurses and midwives whose numbers increased by 370% between 2008 and 2018 [9]. Among the nursing and midwifery workforce, 31% and 22% are enrolled nurses and community health nurses respectively (who are trained for 2 years). Nevertheless, empirically, there exist lingering workforce shortages and inequitable distribution [10, 11] alongside a publicly acknowledged challenge of trained but unemployed nurses and midwives [12, 13]. Although the aforesaid issues continue to preoccupy the health sector policymakers, an examination of the nursing and midwifery demographic transition over the last decade shows a massive youth bulge which must be taken into account in the health workforce policy discourse. Based on the annual operational report of the Ghana Health Service (GHS), we examine the changing age dynamics of the nursing and midwifery workforce in Ghana and advocate for a policy response in terms of mentorship and in-service education management.

## Demographic shift among nurses and midwives—2008 and 2018 compared

**Fig. 1:**
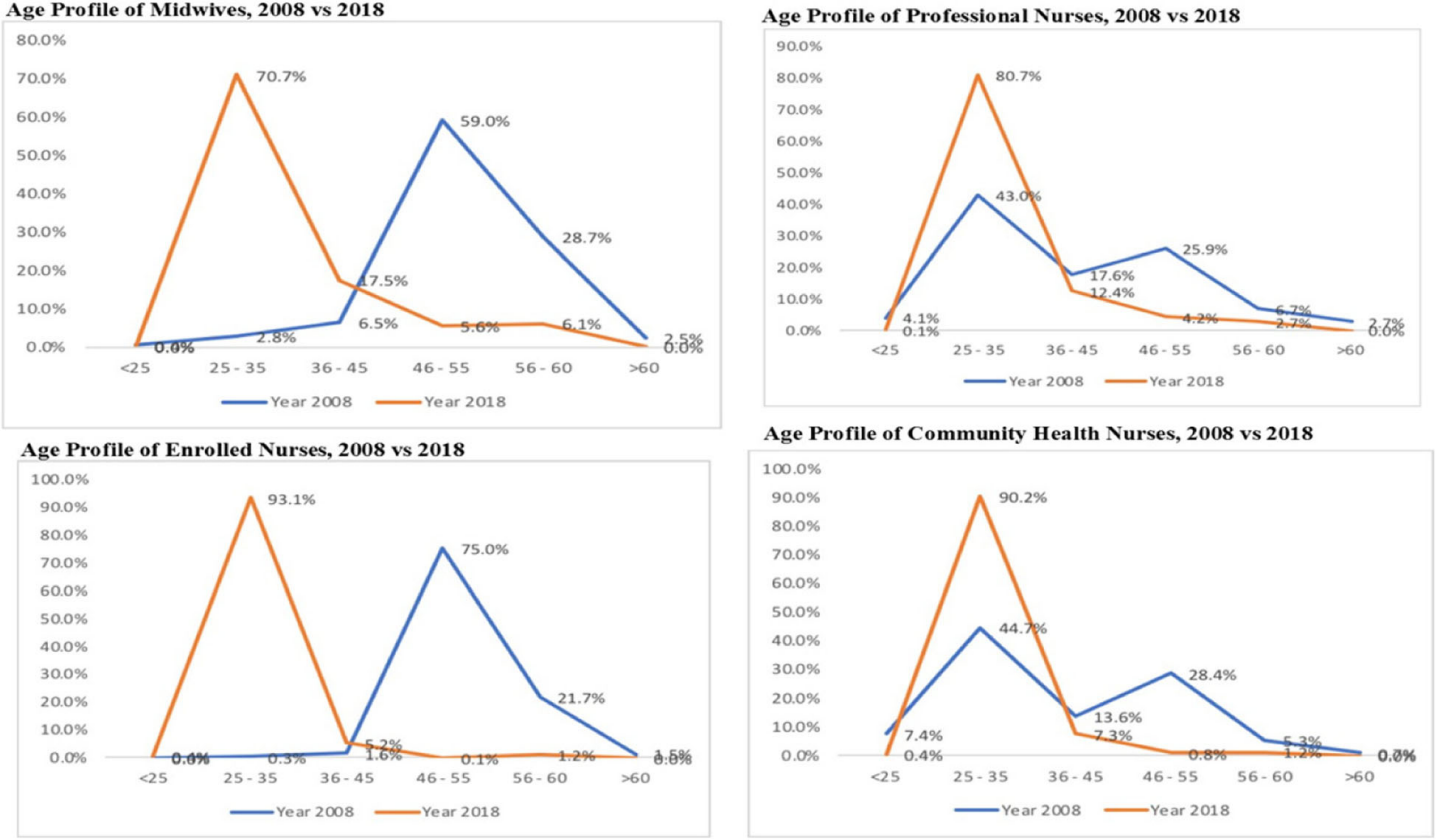
Age profile of nurses and midwives in the public sector of Ghana, 2008 vs 2018

Since the last decade, there has been a far-reaching shift in the age profile of the nursing and midwifery workforce in Ghana which appears to have been unaccounted for in the health workforce policy discourse. For example, in 2008, less than 3% of midwives were younger than 35 years with at least 88% being older than 46 years, most of whom were set to exit the active workforce (Fig. 1). This certainly rang the alarm bell [14] resulting in the introduction of direct Midwifery Program for Secondary School leavers which was hitherto restricted to only serving nurses. In contrast to the 2008 situation, about 71% of midwives in 2018 were between 25 and 35 years. Only 12% of the midwives were older than 46 years compared with 88% in 2008. This implies that during the last decade, there were literally too many experienced midwives with fewer younger ones to mentor, a situation which the opposite is currently prevailing whereby the bulk of the current generation of midwives are relatively inexperienced while there are too few clinical mentors to guide them.

Similarly, in 2008, there were two distinct generational cohorts of professional nurses; 43% of whom were young (25–35 years) and were being mentored by 36% who were aged 45 years or older. Compared to the situation in 2018, 81% of professional nurses are younger than 35 years who are expected to be mentored by only 7% experienced ones who are 46 years or older (Fig. 1).

Also, the pattern of the demographic shift in the ages of the auxiliary nursing categories has been like that of the midwives. By 2018, enrolled nurses and community health nurses who were older than 45 years were almost extinct in the public health sector (less than 3% in both cases), as 93% and 90% of enrolled and community health nurses respectively were younger than 35 years. Indeed, the median range of working experience of these categories of nurses is also 3–5 years.

## Policy implications

The foregoing demographic shift in terms of the age profile of nurses and midwives brings to fore several policy implications, some of which we discuss with the view of advocating for operational policy reforms at the MOH and its agencies.

### Institute nursing and midwifery mentorship programme to improve quality of care

Whilst some have lauded the improvement in the stock of nursing and midwifery workforce, there are also concerns about the quality of nurses and midwives being produced and the impact of same on quality of care [15, 16]. Undoubtedly, a situation where the care of patients is shifted from quite experienced to relatively inexperienced hands should adversely affect the technical quality of patient care, especially when there are no structured clinical mentorship programmes for midwives and nurses in Ghana beyond the required internships which are seldom monitored and virtually no one gets deferred. In the case of midwives, cultural issues may hinder older mothers from delivering in health facilities because they feel the young midwives are of the age of their children. Under the circumstance, it would be imperative for the MOH and its agencies to institute a mentorship scheme for nurses and midwives and if necessary, re-engage retired but fit-to-practice nurses and midwives to mentor young nurses and midwives.

Also, it has been documented that professional nurses of junior ranks (such as staff nurse and senior staff nurse—those with 1–3 years of working experience) have in many instances been made nurse managers in-charge of service delivery units/wards without adequate preparations [17–19]—roles which are significantly supervisory, clinical leadership and mentorship in nature. This ill-preparedness of the nurse managers could have an adverse consequence on both staff and patient outcomes [20–24]. Beyond concerns of the technical quality of patient care associated with limited clinical experience of the young generation of nurses and midwives, another source of concern is the potential for them to get stressed and burnout due to high workload levels in the roles that are relatively above their level of experience and capacity, a situation which could culminate in increased turnover and ultimately higher vacancy rates. Therefore, the Ministry of Health and its agencies need to explicitly define the criteria to be met, preferably competitive and managerial in nature, for appointment as nurse/midwife manager.

### Review in-service training and education policy

It has been observed that, in recent years, large numbers of young health workers especially nurses and midwives are pursuing further training and education because most of them are initially trained at level of a 2-year certificate or a three-year diploma who are seeking for opportunities to obtain bachelor degrees—a testament that many nurses and midwives are unsatisfied with their non-bachelors level of training. Indeed, evidence from the international literature suggests that patients are much more safer under the care of nurses and midwives who are highly trained at least at the level of bachelors [25, 26]. Under the prevailing dispensation of study leave policy, the official opportunities given to nurses and midwives to embark on further studies for bachelor degrees appears to be too restrictive which some nurses and midwives even wonder if it will ever get to their turn if they continue to wait in the queue for study leave. Consequently, some nurses and midwives have resorted to flexible modes of education such as weekends, evening and sandwich studies (with or without approval from their employer) which sometimes put them at the offending side of organisational policies.

It is imperative that Ministry of Health and its agencies view the aforesaid as a clarion call to review the policies on study leave which is neither efficient nor meeting the professional needs of the current generation of nurses and midwives. For instance, in 2018, 67% of GHS’ staff education programme focused on full-time studies with only 32% focusing on weekend, evening and sandwich modes of education combined [9]. The study leave policies should be reviewed to allow flexibility to accommodate the needs of the young generation of nursing and midwifery workforce to eschew resentment and demotivation which is adversely affecting staff performance and service delivery. We advocate for periodic comprehensive training needs assessment of all staff to facilitate the development of a master training plan based on which annual study leave plans can be developed using a bottom-up approach. This should be accompanied with greater emphasis on flexible modes of education (such as weekends, sandwich and evening studies) which tend to be cost-effective from the employer’s perspective as these flexible modes of education would allow the employees to continue to provide services whilst pursuing further studies. Finally, it might be worth considering upgrading all professional nursing and midwifery programmes to the level of first degree (baccalaureate programmes) as is the case in some countries in bid to improve the quality of training and also mitigate against the mass agitations by nurses and midwives for study leave to undertake degree programmes in nursing and midwifery soon after their employment.

## Conclusion

Escalation of the production of nurses and midwives in Ghana over the last decade has resulted in a drastic shift in the age structure of nurses and midwives. This demographic shift appears to have been without appropriate mechanisms for mentorship which could adversely affect the quality of health care. The nursing and midwifery youth bulge has increased pressure on the administration of the study leave policy of the Ghana health sector. We reckon that these have necessitated the institutionalisation of a nursing and midwifery mentorship programme and a review of the study leave policy as well as to consider upgrading all professional nursing and midwifery programmes to first degrees soon after their employment.

## Abbreviations

HRH: Human Resource for Health
HWF: Health Workforce
MOH: Ministry of Health
SDGs: Sustainable Development Goals
UHC: Universal Health Coverage
WHO: World Health Organization
